# Supplementary material for: Are carbon emissions trading and green financial instruments synergistic? -Comprehensive quantitative research based on content analysis
Source: PLoS One. 2024 Mar 7;19(3):e0298601. doi: 10.1371/journal.pone.0298601 (PMC10919601; doi:10.1371/journal.pone.0298601)
Supplement: S1 Data — (ZIP) [file pone.0298601.s001.zip › Data/地级市Z_F工作报告.zip!/╔╜╢1⁄2/▒⌡╓▌╩╨/└┤╫╘╣1⁄2╓┌║┼í╛┬φ┐╦╩2╛▌═°í┐_2011.docx]

各位代表：

现在，我代表滨州市人民政府向大会做工作报告，请连同“十二五”规划纲要（草案）一并审议，并请市政协委员和其他列席会议的同志提出意见。

回顾“十一五” 我们倍感振奋自豪

推动科学发展的信心和决心更加坚定

“十一五”时期，是滨州经济社会持续快速发展，城乡面貌发生巨大变化的时期。五年来，在省委、省政府和市委的坚强领导下，我们抢抓机遇，积极作为，科学实干，有效化解金融危机的冲击，成功战胜各种自然灾害，把保增长、保民生、保稳定等宏观调控政策落到实处，在全面建设小康社会的道路上迈出了坚实步伐。今天，我们可以庄严的回答：经过全市373万人民的共同奋斗，“十一五”的主要指标已经全面完成，一个蓄势待发、充满活力的新滨州正在蒸蒸日上、蓬勃而起！

解放思想、追赶超越，综合实力迅速增强。初步预计，2010年实现地区生产总值1560亿元，人均42100元，是2005年的2倍。实现地方财政收入103.99亿元，是2005年的3.2倍。城镇居民人均可支配收入19686元，农民人均纯收入7194元，分别比2005年增加9400元和3377元。规模以上固定资产投资五年累计完成3166亿元，年均增长22.3%。五年来，我们迎难而上抢机遇，咬定发展不放松，掀起一轮又一轮发展高潮，主要经济指标实现了倍增。

负重奋进、攻坚克难，产业结构加快调整。预计三次产业比例由2005年的13.66∶59.48∶26.86调整到9.62∶55.77∶34.61。努力放大“粮丰林茂、北国江南”的品牌效应，深入实施“林水会战”，五年新增蓄水能力2.25亿立方米，新增合格造林面积86.5万亩，粮食生产连年喜获丰收。农业标准化基地达到165万亩，无公害农产品、绿色和有机食品认证242个。农机化综合水平达到84%。坚持“工业兴市”，2010年规模以上工业实现增加值919.8亿元，比2005年增加574.7亿元；规模以上企业1650家，比2005年增加653家；预计利润、利税分别达到228.9亿元和349.5亿元，分别是2005年的3.5倍和3倍。主营业务收入过百亿的企业9家,比2005年增加7家。高新技术产业产值800亿元，是2005年的8.3倍。全市省级以上企业技术中心32家、中国驰名商标9件、中国名牌产品14个。努力做大做强服务业，预计2010年完成增加值540亿元，比2005年增加363.8亿元。旅游产业快速发展，荣获中国优秀旅游城市称号。面对节能减排特别严峻的形势，面对群众的特别期待，我们不掩盖、不回避，痛下决心、迎难而上，全面完成了“十一五”节能减排目标任务。五年来，我们自我加压，稳中求快，充分运用市场机制和宏观调控政策，实现了经济资源和社会资源的优化配置。

加大投入、强基固本，城乡面貌变化巨大。津汕高速、荣乌高速滨州段建成通车，滨德高速公路滨州段路基工程基本完成，全市新增高速公路102公里。疏港公路全线贯通，新建、改造一级公路295公里。新改造农村公路6000公里。以BOT形式建成黄河上第一座公铁两用大桥，滨港铁路一期开通运营，德龙烟铁路滨州段开工建设。滨州港被纳入全国沿海港口建设布局，11公里引堤工程全部建成，2个3万吨级码头及配套工程加快建设。全力打造“四环五海、生态滨州”，中心城市建成区面积达到83平方公里。成功创建省级园林城市。滨州汽车总站、城市公共供热中心等工程相继投入运营。邮政通讯事业综合指标居全省前列。县城基础设施建设步伐加快，小城镇辐射带动作用逐步增强。全市农村社区达到579个，建设新农村示范村1000个。五年来，面对基础设施建设严重滞后的现状，我们不气馁、不等待，既主动争取上级支持，又集中民智求突破，实施了一大批关乎未来发展的重大项目，为经济社会发展提供了强力支撑。

创新发展、破茧蝶变，改革开放不断深化。市县政府机构改革、财税改革、国企改革、农村综合改革、医药卫生和文化体制改革扎实推进。市财政局获得“全国财政系统先进集体”荣誉称号。多措并举抓招商，累计利用外资11.9亿美元，年均增长17.2%。累计进出口总额190.5亿美元，其中出口100.8亿美元，年均增长17.2%和15.5%。累计争取国家财政性支持资金37.1亿元。银企合作成效显著，累计签约资金5795亿元，到位率达到92.6%，银行存贷款余额双双跨过千亿元大关。邮政储蓄银行、齐商银行、东营商行、浦发银行、恒丰银行等5家商业银行在我市设立机构。小额贷款公司发展到15家，在全省率先实现县区全覆盖。境内外上市公司11家，比2005年增加6家。保险公司发展到25家。山东滨州国家农业科技园区获科技部审批。高新技术产业开发区、北海经济开发区相继设立，省级以上经济开发区达到10家。滨州经济开发区荣获全省对外开放先进园区，邹平经济技术开发区成为国家级开发区。邹平县连续七年、博兴县连续两年进入全国百强县。五年来，我们坚持向改革要出路，向改革要效益，向改革要发展，不断拓展对外开放的广度和深度，发展活力持续迸发。

关注民生、统筹协调，和谐局面更加巩固。社会事业五年累计投入265亿元，是“十五”期间的近3倍。深入开展“解决城乡家庭零就业年”活动，新增城镇就业20.5万人，转移农村劳动力45万人次。社会保障体系不断完善，建立城镇居民基本医疗保险制度，扎实推进新型农村养老保险试点工作，城乡低保、五保救助水平显著提升。市社会福利中心、儿童福利院建成。解决和改善了179万农村居民的饮水安全问题。建设经济适用房、廉租房、公共租赁住房109.5万平方米，完成农房建设、危房改造10.8万户。科技创新能力不断提高，被评为全国科技进步先进市。素质教育全面推进，教育质量稳步提升，义务教育实现全免费，教育公平迈出重大步伐。高等教育、职业教育健康发展，滨州职业学院争创为国家骨干职业院校建设单位。公共文化四级网络基本形成，连续两届荣获全国精神文明建设“五个一”精品工程剧目奖，渤海革命老区纪念园等文化基础设施建成使用。市文化中心工程局部封顶。新型农村合作医疗参合率达到100%。成功承办了十一届全运会武术套路和柔道两项赛事，竞技体育水平跃居全省先进行列。军民共建成效显著，被评为“全国双拥模范城”。人口计生工作连年获得省考核最高奖。加强精神文明建设、民主法制建设和社会治安综合治理，深入开展严打整治斗争，平安滨州建设成效显著。安全生产形势稳定好转。文化市场执法、民族宗教、妇女儿童和国土、物价、档案、史志、人防、外侨、对台、老龄、防震、气象、水文、海事、红十字、残联、慈善、油区管理等各项事业都取得新成绩。特别是在突发事件和重大自然灾害面前，全市上下众志成城，有效防控了甲型H1N1流感和手足口病等重大传染性疾病的暴发流行，出色地完成援川任务，尤其在特大洪水灾害面前，科学指挥，实现了人员无伤亡的可喜战果。五年来，我们始终坚持以人为本，始终关注百姓的“衣食住行”、“生老病残”、“教科文卫”，实现了人民群众对发展成果的充分共享。

依法行政、提速增效，行政效能大幅提升。坚决贯彻市委各项决策部署，自觉接受人大依法监督和政协民主监督。加强依法行政，深化行政审批制度改革，许可事项集中审批比例从16%提高到76%。电子政务建设跻身全省前列。受理市长公开电话30.2万件、市长信箱来信5000件，受理率和办结率分别达到99.2%、100%。强化监察和审计监督，加大行政问责力度，反腐倡廉建设取得新成效。五年来，我们持续全面地加强政府自身建设，精心打造服务政府、责任政府、法治政府，推行“阳光政务”，推进信息公开，行政效率明显提速。

各位代表!“十一五”时期取得的巨大成就,揭开了滨州发展的辉煌一页。这些成绩的取得，是省委、省政府和市委坚强领导的结果，是广大干部群众辛勤劳动的结果，是社会各界全力支持的结果。在这里,我代表市人民政府,向为滨州发展做出贡献的老领导、老同志，向辛勤工作在各条战线上的全市人民,向各民主党派、工商联、无党派人士和社会各界,向中央、省驻滨单位,向驻滨人民解放军和武警官兵,向所有来滨的投资者、劳动者和关心支持滨州发展的海内外朋友们，致以崇高的敬意和衷心的感谢!

回眸“十一五”走过的历程，我们清醒地认识到，当前还面临不少困难和问题。主要是：经济总量小、底子薄、基础差的局面还没有根本扭转；经济结构不合理，服务业支撑作用较弱，自主创新能力不强，城乡区域发展不协调；发展方式粗放，资源、能源、环境约束更加凸显，节能减排面临新的压力和挑战；基本公共服务均等化水平亟待提高；农民持续增收压力较大，涉及群众切身利益的问题还没有得到很好解决，尤其是城市供热、公共交通、环境污染、子女入园、上学等问题反映比较突出；制约科学发展的体制机制障碍依然较多；政府自身建设还需要加大力度，作风建设有待进一步加强。对这些问题，必须以高度负责的精神，采取更加有力的措施，切实加以解决。

五年辛勤耕耘，五年春华秋实。五年来，全市人民用勤劳的双手和创业的激情，谱写了一曲追赶超越的壮丽篇章。在这场伟大生动的实践中，我们付出了艰辛、收获了希望，积累了经验、磨炼了意志，提升了境界、锻造了队伍，进一步坚定了全市上下科学发展的决心。我们坚信，只要我们创造性地贯彻中央宏观经济政策和省委、省政府的重大决策部署，就一定能够持续快速发展；只要我们立足市情定目标，坚持发展不松懈，就一定能够加快追赶超越；只要我们坚持民生优先，就一定能够实现社会的和谐稳定；只要我们同心谋事、合力共为、知难而上、敢于胜利，就一定能够创造出更多奇迹，一定能够创造出更加灿烂辉煌的明天！

展望“十二五” 我们倍感使命光荣

推动科学发展的激情和斗志更加旺盛

从今年开始，我们已经进入了“十二五”时期。站在这个新的起点上，我们既心潮澎湃、豪情满怀，又深感任重道远、责任重大！

“十二五”时期是我们全面建设小康社会、推动科学发展的关键时期，是加快转变经济发展方式、实现追赶超越的攻坚时期，我们要高举中国特色社会主义伟大旗帜，以邓小平理论和“三个代表”重要思想为指导，深入贯彻落实科学发展观，抢抓黄河三角洲高效生态经济区和山东半岛蓝色经济区建设机遇，坚持“科学发展是主题，保障民生是根本，调整转型是主线，招商引资是重点，创新实干是关键，和谐稳定是基础，清正廉洁是保证”的工作方针，按照“123456”的总体发展布局，深入实施追赶超越战略，加快构建高效生态产业体系，改善社会民生事业，统筹城乡一体发展，完善基础设施建设，加强生态环境保护，深化改革开放，努力建设富裕文明、和谐幸福的现代滨州。主要预期目标是：到2015年，全市地区生产总值力争超过3000亿元，地方财政收入力争超过200亿元，城镇居民人均可支配收入、农民人均纯收入分别超过36000元和13000元，实现地区生产总值、地方财政收入、劳动者收入“三个翻番”；固定资产投资五年累计7000亿元；三次产业结构调整为6：52：42；城镇化率达到51%；林木覆盖率达到28.9%；人口自然增长率控制在5.5‰以内；化学需氧量、氨氮排放量分别削减12%，二氧化硫、氮氧化物排放量分别削减18%。

贯彻这一指导思想，实现这个奋斗目标，需要发挥优势、挖掘潜力，需要扬长避短、乘势而上，需要付出百倍的艰辛和努力。历史和现实告诉我们，目前滨州已形成“科学发展、追赶超越”的“六大优势”。千载难逢的战略优势：黄河三角洲高效生态经济区、山东半岛蓝色经济区建设上升为国家战略，“两区”同步开发的机遇效应将不断放大。天时地利的区位优势：“两区”、“两圈”在我市叠加交汇，具有深化国际国内区域合作、加快开放开发的有利条件。多元优质的资源优势：渤海、黄河，平原、山区，以及土地、矿产、滩涂等优势互补的资源，为错位发展提供了巨大的创新空间。初具规模的产业优势：支柱产业高峰耸立，骨干企业快速膨胀，特色产业蓬勃发展，区域竞争的综合实力更加强大。特色鲜明的企业家优势：多年的大浪淘沙，培养造就了大批优秀管理团队，企业家队伍不断壮大，形成了“梯队结构”。厚积薄发的文化优势：黄河文化、齐文化的厚重底蕴凝结历史、连接未来，传承老渤海精神，弘扬新滨州精神，将成为我们续写辉煌的不竭动力。

站在滨州看滨州，我们已经走出“昨天”落后的阴影和困扰，正在追赶超越的征程上昂首阔步；跳出滨州看滨州，我们没有任何理由沾沾自喜，面对区域竞争的巨大压力，要敢于承接更要积极应对；着眼未来看滨州，各项事业需要加快推进、整体提升，任务艰巨但大有可为。尤其是发展高效生态经济和蓝色经济是一项崭新的事业，没有现成经验可循，按照老的工作套路，不可能有质的突破，必须解放思想，找准坐标，用足用好用活政策，把优势和潜力充分发挥挖掘出来。一是必须提升思想境界，突出科学发展这个主题。从“发展”到“科学发展”，是经过几十年探索实践、总结各方面经验教训、准确分析把握国内外大势得出的重要论断。这既是时代的要求，更是滨州自身发展的迫切需要。我们必须牢固树立、坚定不移，必须认真践行、毫不动摇，真正内化为理念转变，外化为自觉行动。二是必须坚持高效生态，突出调整转型这条主线。“转方式、调结构”是“十二五”时期必须破解的重大课题。当前，发展新型工业时不我待，传统农业向现代农业转变迫在眉睫。“如何转、转什么、怎么转”，亟需各级作出回答。“三个翻番”并不是粗放式增长，而是要进一步明确主攻方向，把握“转”、“调”重点，闯出一条资源节约型、环境友好型的高效生态发展之路。三是必须强调创新实干，突出奋发有为这个标准。一切办法，唯有创新实干才能找到；一切问题，唯有创新实干才能解决；一切机遇，唯有创新实干才能抓住。要大力弘扬求真务实之风，创新发展思路，勇破发展难题，奏响发展强音，努力有所作为。四是必须注重社会效益，突出保障民生这个根本。“求木之长，必固其根。欲流之远，必浚其源”。经济社会发展的最终目的，是提高人民群众的幸福指数，谋求人民最大的福祉。今后五年，将大幅度增加民生投入，倾力改善民生，增加城乡居民收入，让发展的巨大成果发挥出最大效益，让全市人民生活得更加幸福、更加安康、更加美好！

“十二五”期间，突出抓好六个方面的战略任务。

(一)着力构建现代产业体系。产业结构调整既充满挑战，又刻不容缓。要按照高效生态的要求，突出农业的基础地位，努力构建优质粮棉、优质蔬菜、生态畜牧等七大现代农业产业体系，扎实推进农业产业化、标准化、规模化，加快由传统农业向现代农业转变。突出环境友好型工业的支柱地位，着力打造家纺服装、新型生态化工、先进装备制造、绿色食品深加工等四个产业基地，培育发展新材料、新能源、电子信息等战略性新兴产业，支持发展中小企业，推进工业化和信息化深度融合。规模以上工业增加值达到1600亿元；主营业务收入过10亿元的企业比2010年增加1倍以上，突破100家。突出服务业的优势地位，优先发展现代物流、金融保险、法律服务等生产性服务业，改造提升旅游、商贸等生活性服务业，培育发展文化创意、服务外包等新兴服务业，积极打造环渤海西南岸物流和商贸中心、黄河三角洲金融服务中心，大幅度提升服务业在国民经济中的比重。突出50个特色园区的载体地位，引导同类和关联产业集中集约集聚发展。坚持陆海统筹，加快培育海洋生物制药、海洋装备制造、海洋化工、海水综合利用、现代海洋渔业等产业，大力发展港口物流业，建立完善现代海洋产业体系。

(二) 着力提高基础设施支撑能力。坚持基础设施先行，是滨州加快发展的基础和保障。要适应“两区”建设的新要求，加快港口、铁路、公路、机场等重大交通基础设施建设，力争到2015年，滨州港吞吐能力达到2000万吨，全市铁路里程达到450公里，国省道干线公路1600公里，形成便捷、高效、安全的综合运输网络。加快建设山东省重要的清洁能源基地，积极稳妥地发展风电、垃圾焚烧发电、生物质能、太阳能等可再生能源。加快水利设施建设，提高水资源供给、水灾害防御和水生态保护三大安全保障能力，全市一次性蓄水能力超过10亿立方米。

（三）着力发展社会事业和保障改善民生。以完善基本公共服务体系建设为目标，深入实施“科教兴滨”和“人才强市”战略，加强产学研合作，全面提高自主创新能力。深入推进基本公共服务均等化，优先发展教育事业，深化教育教学改革，全面推进素质教育，完成标准化学校建设任务，促进基础教育、职业教育、高等教育科学发展。着力发展文化事业、文化产业，加快发展卫生、人口计生、体育事业。努力推动养老服务社会化。深入推进社会保障体系建设，每年新增城镇就业3.5万人，新增农村劳动力转移就业5万人，不断提高社会保障水平，让群众共享改革发展成果。深入开展平安文明建设，营造民主团结、公平正义、和谐稳定的社会环境。

（四）着力推进城乡统筹协调发展。进一步提升城乡规划建设管理水平，积极推进中心城区、县域中心城市、小城镇建设，构建两条贯穿黄河两岸的城市发展轴线，打造新型城镇化体系。积极提升“四环五海、生态滨州”建设水平，完善提升城市交通、供排水、气热供应、信息网络系统，努力增强中心城区城市功能，打造黄河三角洲中心名城。积极发展县域经济，发挥邹平、博兴带动作用，突破阳信、惠民，集中培育“四大发展亮点”，促进县域经济平衡发展。围绕“粮丰林茂、北国江南”目标定位，继续深化林水会战，推进农村新型社区建设，加快社会主义新农村建设。

（五）着力加强生态建设和环境保护。加快建设生态文明滨州，是推进黄河三角洲开发建设的必然要求。要正确处理推进工业化、城镇化与建设生态文明之间的关系，强力推进生态建设，合理构建以农田、林带、水系、湿地为主的生态格局。强力推进环境保护和水、大气等污染治理，切实提高环境承载能力。强力推进循环经济企业和生态工业园区发展，加快建设全国重要的循环经济示范基地。强力推进节能减排，积极发展低碳经济、绿色经济、清洁生产。强力推进水、土地、海域集约节约利用，建立完善资源开发保护的长效机制。

（六）着力深化改革扩大开放。加快经济发展方式转变，既是一场攻坚战，也是一场持久战，必须通过深化改革、扩大开放来推动。要着眼于增强追赶超越的动力和活力，以更大的决心和勇气推进行政管理体制、投融资管理体制、土地管理体制等各领域的改革，先行先试，大胆探索，重点突破。以更大的魄力和干劲加快对外开放进程，进一步完善对外开放的平台，提高招商引资的质量和水平，转变外贸发展方式，充分利用好国际国内两个市场、两种资源，在更广领域和更高层次上融入全国、全球经济发展的大格局。

站在新的起点上，能不能抓住历史机遇，战胜风险和挑战，很大程度上取决于我们的精神状态。我们一定要牢记使命，不负重托，再接再厉，砥砺奋进，努力谱写今后五年科学发展的新篇章！

着眼2011年	我们倍感任务艰巨

推动科学发展的责任和压力更加紧迫

2011年是“十二五”的开局之年，山东半岛蓝色经济区开发的起步之年，更是我市黄河三角洲高效生态经济区建设的全面深化之年。新的一年里，我们将拼搏创新，务实苦干，深入开展“发展环境提升年”、“重点项目突破年”、“质量效益提高年”活动，全面掀起“两区”开发建设的新高潮。主要预期目标是：全市地区生产总值实现1780亿元，增长14％；地方财政收入实现119.6亿元，增长15％；规模以上固定资产投资完成1070亿元，增长21％；规模以上工业增加值实现1048亿元，增长14％；外贸进出口总额达到58.5亿美元，增长15％；城镇居民人均可支配收入、农民人均纯收入分别达到22245元和8130元，均增长13％；居民消费价格涨幅控制在4%左右；城镇登记失业率控制在3.6％以内；人口自然增长率控制在5.5‰以内；万元GDP能耗下降3.66%；化学需氧量、氨氮、二氧化硫、氮氧化物排放量分别削减2％。着重抓好六个方面工作。

（一）全力以赴打好转方式调结构硬仗。大力发展高效生态农业。立足资源条件和产业基础，打造黄河三角洲高效生态农业示范区、绿色农产品基地、国家级农业园区三大品牌，加快构筑优质、高产、安全的高效生态农业产业体系。规模以上农业龙头企业新增27家，达到520家。农业标准化基地新增15万亩以上，达到180万亩。重点培植58个高效生态农业示范园区。抓好黄河三角洲国家级现代畜牧业园区和水产健康养殖园区的规划实施。加快山东滨州国家农业科技园区建设，努力打造中国农业物联网中心。积极落实强农惠农政策，推动农村非农产业发展，促进农民稳步增收。

集中推进工业调整提升。加大企业技术改造力度，加强项目的储备和引领，重点抓好340个投资过千万元的工业技改项目。强化产业关联度，突出集约化，推动机械加工等产业向上游延伸，纺织、化工、粮油加工等产业向中下游延伸，着力发展终端、高端产品，推动传统产业转型升级。依托重点行业和重点区域，实施一批重大信息化与工业化融合项目。聘请高层次专家筛选论证一批重点项目，加快科技成果转化，推动新兴产业提高核心竞争力。大力支持企业技术创新，引进和培养一线创新技术人才，增强企业科技研发竞争力，全年完成技术创新项目1500项。全面推进企业管理创新，实施名牌带动战略。大力发展中小企业，搞好金融、担保、科技、培训等服务体系建设，组建并发挥行业协会的作用，大力培植龙头企业，打造各具特色、分工协作、差异化发展的特色产业集群。

推动服务业跨越发展。抓好重点城区、重点园区、重点企业、重点项目“四大载体”建设，全市服务业占生产总值比重提高1.5个百分点。以打造鲁北物流中心为目标，整合物流资源，加快临港物流园区、黄河三角洲铁路物流园区、京博物流中心等重点项目建设。改造提升商贸流通业，提高城乡市场、商业网点建设水平，培植年销售收入过10亿元的商贸流通企业2家，发展交易规模过20亿元以上的商品交易市场8家。促进商品流通规范发展，引导热点消费需求，让群众有能力消费、有地方消费、安全放心地消费。推动旅游与文化的深度结合，抓好黄河岛、兴国寺佛教文化园等十大重点旅游项目，推进贝壳堤、三河湖旅游度假区建设。健全文化市场中介机构和行业组织，扶持发展文化创意、现代传媒、节庆会展等文化产业。加快金融产业发展，强化银企合作，构建起多方位、立体式的金融服务体系。鼓励企业加快非核心业务剥离，确保服务业增加值增长17.5%。

加快发展海洋经济。积极对接山东半岛蓝色经济区发展规划，研究制定实施意见，科学规划产业布局，争取更多项目获得国家、省倾斜支持。以无棣、沾化开发为重点，以政策创新为保障，强力推进相关工作和项目实施。其他县区作为联动区统一规划，主动对接，配套发展，确保年内取得实质性突破。

打好节能减排攻坚战。加快城镇污水处理厂改造提升步伐，日处理能力达到51.5万立方米。严格监管67家省控重点污染企业，确保流域水质稳定达标，确保在国家海河迎查中取得优异成绩。组织实施循环经济示范工程，加快推广节能新技术、新工艺、新产品，突出抓好建筑、交通和公共机构节能，淘汰落后产能。大力发展循环经济，积极推行清洁生产，确保完成省下达的节能减排目标任务。

（二）全力以赴加快基础设施建设。增强交通运输综合承载力，加快滨德高速以及大济路、庆淄路、广青路、博临路等续建路网项目建设，确保年内竣工通车。推进济南—滨州—东营高速公路、沿海高等级公路的前期工作，力争年内开工建设。启动东青高速连接线前期工程。确保滨州港2个3万吨级码头年内实现简易运营。推进德龙烟铁路滨州段加快建设，力争黄大铁路、寿邹铁路开工建设。完善北海经济开发区水电路讯等基础配套设施，尽快拉开发展大框架。

创建“国家级园林城市”，高起点、高标准编制城市重点区域控制性规划，构建科学的城市空间发展格局。依托两条城市轴线，搞好各县区规划的衔接配套。进一步做大做强中心城市，计划安排市本级城建项目78项，总投资25.3亿元，突出抓好集中供热、城市道路及城区28个城中村改造等重点工程。加强园林绿地建设，完善城市数字化管理，以人为本做好城管执法。加快县城建设步伐，配套建设村镇基础设施及公共服务设施，有序推进符合条件的农村转移人口落户城镇。

强化农村基础设施支撑。继续抓好林水会战，建设标准化方田22万亩，完成各类水利工程464项，抓好5个“全国小型农田水利建设重点县”项目实施。完成成片造林17万亩、绿色通道精品工程272公里。抓好沾化秦口河、博兴麻大湖等生态湿地修复和保护工程建设。强化河道治理，推进大中型灌区续建配套与节水改造。配合搞好南水北调、胶东调水工程。保护性耕作面积达到100万亩，覆盖率达20%以上。完善农村交通网络，新建改建农村公路400公里以上。加快推进新一轮农村电网升级改造。严格落实县域村镇体系规划和土地利用总体规划，积极稳妥地推进农村土地综合整治，加快建设农村新型社区。

（三）全力以赴推动改革开放。完成县级政府机构改革任务，搞好“强镇扩权”试点。积极应对中央和省财税、金融、投资和资源环境等领域的政策变化，建立完善县级基本财力保障机制。加快事业单位改革，推进政事职责分开。健全完善国有资产监督管理体制，认真抓好公司制股份制改革。启动推进公立医院改革和国家基本药物制度试点，确保完成省政府下达的医改任务。完善乡镇“三农服务中心”建设，依法、自愿、有偿搞好土地流转，提高节约集约用地水平。

进一步扩大对外开放。大力实施产业兴贸、科技兴贸、品牌兴贸、市场多元化四大战略，加快进出口产业结构调整，积极开拓国际市场，新增进出口企业100家。围绕重点产业发展规划，瞄准重点地区集中招商，加强与世界500强和央企的战略合作。积极筹备好第二届黄河三角洲高效生态经济区经贸洽谈会等大型经贸活动。积极推动企业上市工作，争取年内2家企业在境内外首发上市。支持企业加快“走出去”步伐，拓展发展空间。

强力推进园区建设。落实产业规划，强化功能定位，配套完善基础设施，进一步增强综合承载功能和辐射带动能力。按照项目园区化发展方向，推动企业进区入园、关联发展，延伸拉长产业链，提高单位土地投资强度和产出效益，推进资源综合利用，加快形成高效生态产业体系，打造新的经济增长极。

加快区域经济协调发展。大力提升邹平、博兴经济发展质量，加大突破阳信、惠民工作力度，培植壮大无棣、沾化优势产业，推动滨城区提高服务业发展水平。举全市之力开发建设北海经济开发区，优化滨州经济开发区和高新技术开发区产业布局。

（四）全力以赴加快和谐社会建设。坚持“科教兴滨”战略。创新产学研合作模式，鼓励自主创新，引导高校、科研院所通过技术入股等形式，参与企业技术改造及发展高新技术产业。强力推进校舍安全工程和学校标准化建设，着力改善农村中小学办学条件。大力发展学前教育，深入实施素质教育，全面落实“以县为主”管理体制和教育惠民政策。大力支持职业教育、高等教育建设。

加快发展人口计生和卫生事业。全面提升医疗水平和服务质量，有效防控重点传染病的暴发流行。积极推进市公共卫生中心主体工程建设。统筹解决人口问题，圆满完成省政府下达的责任目标。

繁荣发展文体事业。完善公共文化服务体系，引导城市文化资源向农村流动。建成市文化中心并投入使用。开工建设“一馆三中心”。全面完成奥林匹克公园一期工程。搞好市奥林匹克体育馆等大型场馆设施的市场化运营，推动全民健身活动，不断提升竞技体育、群众体育水平。

广泛组织文明创建活动。深入开展普法教育，强化依法治理。扎实做好双拥共建工作。加强社会治安综合治理和社会管理创新，依法管理文化市场，严密防范、严厉打击各类违法犯罪活动。严格安全生产监督管理。加强应急管理，妥善处置各类突发事件。做好民族宗教工作，促进民族团结进步和宗教和谐。

（五）全力以赴保障和改善民生。全面落实积极的就业政策。完善社会保障体系，扩大社会保险覆盖范围。加快推进新型农村养老保险制度试点。积极落实优抚政策，大力发展慈善事业。建立健全城乡困难群众社会救助体系。落实各项扶残助残优惠政策。逐步建立以财政为主的农村五保供养机制，集中供养率稳定在80％以上。在此基础上，大力实施“十大民心工程”。

就业创业工程。实施创业带动就业战略，县区普遍建立现代农业、工业、服务业就业创业园区和创业孵化基地、高校毕业生见习基地。每个县区重点抓好1—2处创业园区，带动就业6000人以上。

教育文体工程。完善深化义务教育经费保障机制，提高农村中小学校生均公用经费水平。资助家庭困难学生4.7万人。提升乡镇文化站、村文化大院建设，文化信息资源共享规范化基层服务站点达到40%。新建农家书屋279家。市县城区数字电视实现全覆盖。加强全民健身公共场所建设。

民生水利工程。新增和恢复蓄水能力4127万立方米。解决和改善13.5万农村居民的饮水安全问题。搞好防潮堤建设，推进城乡防汛排水工程和病险水库除险加固建设。实施渤海七路改造、黄河一路道路排水改造等工程，进一步提高城市防汛设施的承载能力。

环境优化工程。加快水系截污工程建设进度，强化水气环境整治和监管。城区生活垃圾无害化处理率达到100%。继续开展国家级和省级生态乡镇建设，推进农村环境综合整治。

健康服务工程。强化基层医疗卫生服务机构能力建设，优化中医药发展环境，加快卫生信息化建设步伐。新创建标准化基层医疗卫生服务机构200处，城乡居民健康档案建档率达到98%，实现新农合规范管理。

放心食品工程。推进社区便民超市建设，培育便民超市连锁经营企业，建立农副产品物流仓储、配送体系，严格质量安全检测。开展“放心粮油”进城下乡服务活动，设立服务网点3000个，创建放心粮油加工示范企业10个。

安居保障工程。建设廉租住房1600套、经济适用住房1240套、公共租赁住房2700套。新建农房2万户，全面完成危房改造任务。

社会养老工程。加快推进以居家养老为基础、社区服务为依托、机构养老为补充的社会化养老服务体系建设。设立5处养老机构示范点，新建3处县级社会福利服务中心，开展老年人社区日间照料和居家养老服务示范工作。

公交便民工程。优化公交线网布局，新设立公交线路5条，延长线路12条，城市公交日综合运量达到7万人次。新投放清洁能源公交车100辆以上，逐步构建低碳公交系统。

城市供暖工程。积极推进市城区集中供热设施及节能改造，加快供热管网建设与改造，确保集中供热面积达到750万平方米，力争城市集中供热普及率达到80%，供暖效果和服务质量有明显提高。

（六）全力以赴推动政府自身建设。强化依法行政，健全完善重大问题集体决策、专家咨询、法律顾问、网络听政等科学民主决策机制。提高电子政务水平，实现政府监管和公共服务基础信息共享。建设综合电子监察平台和公共资源交易中心，深入推进全市机关一网式协同办公。强化“公开是原则，不公开是例外”的理念，进一步推进政府信息公开，增强政府工作透明度和公信力。扩大行政审批网上办理和市县两级联动审批。认真办理人大议案、代表建议和政协提案，自觉接受人大及其常委会的依法监督和人民政协的民主监督。深化机关效能建设，加大绩效考评力度。创新督查方式，严格行政问责。扎实推进惩治和预防腐败体系建设。加强政府系统干部队伍建设，努力塑造政治坚定、爱岗敬业、廉政为民的良好形象。

各位代表！宏伟蓝图已绘就，奋发有为正当时。伴随着“十二五”规划的全面实施，一个新的征程已经开启。让我们在市委的坚强领导下，深入贯彻落实科学发展观，进一步解放思想，抢抓机遇，锐意进取，攻坚破难，以更加优异的成绩迎接建党90周年，为建设富裕文明、和谐幸福的现代滨州而努力奋斗！
